# Independent Component Analysis-motivated Approach to Classificatory Decomposition of Cortical Evoked Potentials

**DOI:** 10.1186/1471-2105-7-S2-S8

**Published:** 2006-09-26

**Authors:** Tomasz G Smolinski, Roger Buchanan, Grzegorz M Boratyn, Mariofanna Milanova, Astrid A Prinz

**Affiliations:** 1 Department of Biology, Emory University, Atlanta, Georgia, USA; 2 Department of Biology, Arkansas State University, Jonesboro, Arkansas, USA; 3 Kidney Disease Program, University of Louisville, Louisville, Kentucky, USA; 4 Computer Science Department, University of Arkansas at Little Rock, Little Rock, Arkansas, USA

## Abstract

**Background:**

Independent Component Analysis (ICA) proves to be useful in the analysis of neural activity, as it allows for identification of distinct sources of activity. Applied to measurements registered in a controlled setting and under exposure to an external stimulus, it can facilitate analysis of the impact of the stimulus on those sources. The link between the stimulus and a given source can be verified by a classifier that is able to "predict" the condition a given signal was registered under, solely based on the components. However, the ICA's assumption about statistical independence of sources is often unrealistic and turns out to be insufficient to build an accurate classifier. Therefore, we propose to utilize a novel method, based on hybridization of ICA, multi-objective evolutionary algorithms (MOEA), and rough sets (RS), that attempts to improve the effectiveness of signal decomposition techniques by providing them with "classification-awareness."

**Results:**

The preliminary results described here are very promising and further investigation of other MOEAs and/or RS-based classification accuracy measures should be pursued. Even a quick visual analysis of those results can provide an interesting insight into the problem of neural activity analysis.

**Conclusion:**

We present a methodology of classificatory decomposition of signals. One of the main advantages of our approach is the fact that rather than solely relying on often unrealistic assumptions about statistical independence of sources, components are generated in the light of a underlying classification problem itself.

## Background

Signals recorded from the surface of the cerebral cortex are composites of the electrical activity of a large number – probably millions to billions – of individual cells. Therefore, one would expect that several different processes – each produced by a different neuronal structure with a characteristic activity pattern – would be occurring simultaneously. The critical question here is: Can these superimposed signal patterns be separated and analyzed independently? In order to address that issue, we propose to utilize an experimental technique based on measuring neural activity in a controlled setting (normal) as well as under exposure to some external stimulus – nicotine, in this case [1]. Application of stimuli that affect the observed signals often has an effect only on a subset of the sources. The information about which sources are affected by the stimuli can provide an interesting insight into the problem of neural activity analysis, but cannot be measured directly. Based on the assumption that each of the sources produces a signal that is statistically independent of the others, the observed signals can be decomposed into constituents that model the sources, also referred to as basis functions. Each of the observed signals is a linear combination of those modeled sources. Due to the fact that some sources influence some locations stronger than others, each source can be present in each observed signal with a different magnitude. The magnitudes are modeled as coefficients in the aforementioned linear combination. The change in the coefficients, as a result of applied stimuli, corresponds to the change in the contribution of a source in generation of a given signal.

Independent Component Analysis (ICA) can be useful in this kind of analysis, as it allows for determination of an impact of the external stimuli on some specific neuronal structures, supposedly represented by the discovered components. The link between the stimulus and a given source can be verified by a classifier that is able to "predict" under which condition a given signal was registered, solely based on the discovered independent components.

The general idea behind all decomposition techniques is to represent the original signal **x **in terms of some basis functions **M **and a set of coefficients **a**, with an addition of some noise or, simply, error **e**:

**x **= **Ma **+ **e**.       (1)

With this approach, the temporal properties of the system are preserved by the basis functions. The original sequences are replaced by a set of scalar coefficients that represent the original data in the space spanned by the basis functions. The process of reconstruction into the original input space is simply based upon a linear combination of the basis functions *(i.e*., a sum of the basis functions weighted by the coefficients).

**Figure 1:**
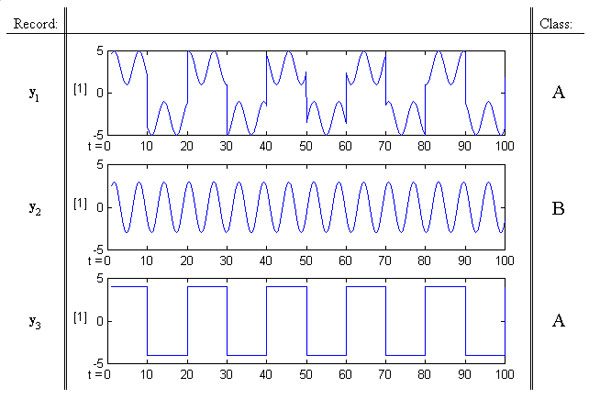
Signal decomposition for classification – original dataset (source: [2])

**Figure 2:**
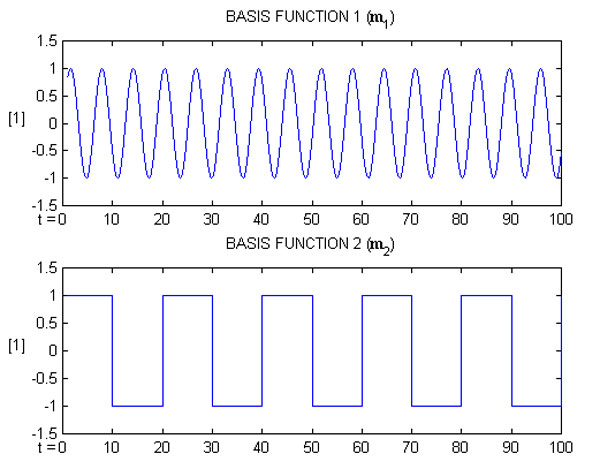
Signal decomposition for classification – generated basis functions (source: [2])

**Figure 3:**
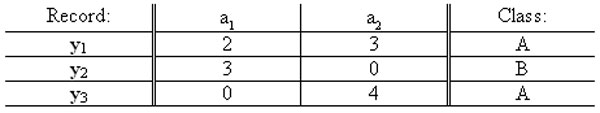
Signal decomposition for classification – coefficients for representation of the original signal in new attribute space (source: [2])

For example, the following artificially generated dataset consisting of three sequences **y**_1_, **y**_2_, **y**_3 _belonging to one of the two categories A and B each (Fig. 1), can be replaced by two basis functions **m**_1_**, m**_2 _(Fig. 2) and a new dataset consisting of the coefficients a_1_, a_2_, (Fig. 3), for the basis functions **m**_1_, **m**_2 _respectively, that will represent the original vectors **y**_1_, **y**_2_, **y**_3 _in the new attribute space [2].

Such a transformation can be very useful for classification. The feature space has been tremendously reduced *(i.e*., instead of operating on vectors with 100 values each, just two numbers are being used) and the task becomes straightforward – in this example, even a single decision rule will be sufficient to classify the signals in the database without error.

In the above example, the signals of class A are those that contain both the sinusoidal and the square pulse components, while the type B sequences have no square component at all. This could possibly be deduced from the analysis of the shapes of the signals, but can also be based upon the analysis of the coefficients and some simple classification rule generation *(e.g*., IF a_2 _is 0, THEN Class is B, ELSE Class is A). This is a very simple, synthetic example, so the classes are known *a priori; *however, one can imagine a "real-life" problem where such a solution would be very desirable. For example, assuming that signals in class B are registered right after an application of some external stimulus, one could conclude that the stimulus inhibits the source that generates the square pulse, but has no apparent influence on the source related to the sinusoidal component.

Over the years, various decomposition techniques have been successfully applied to the domain of signal classification. Unquestionably, one of the most commonly used methods for that task is Independent Component Analysis (ICA). Even though it proves to be a powerful and often successful tool, one of the main weaknesses of ICA is its assumption about the statistical independence of the components – this will rarely be sufficient for a successful differentiation between signals that belong to different classes. Another important flaw of ICA is the fact that the cardinality of the resultant set of independent components is always the same as the number of the input signals. This poses a difficult question as to the importance of the discovered components for a given classification task: which of the components explain the classification in the best possible way? This can also become a major computational problem, especially with a large size of the analyzed database. Thus the idea of combining the premises of a reliable and accurate decomposition of signals (verifiable via the reconstruction process) with the determination of the components that really matter in terms of segregation of the input sequences into separate categories seems plausible.

Classificatory decomposition (CD) is a general term that describes our research study that attempts to improve the effectiveness of signal decomposition techniques by providing them with "classification-awareness". The description of previous stages of the study and some examples of applications can be found in [2-5]. In this article, we investigate hybridization of multi-objective evolutionary algorithms (MOEA) and rough sets (RS) to perform decomposition in the light of the classification problem itself. The idea is to look for basis functions whose coefficients allow for an accurate classification while preserving the reconstruction. We propose a simple extension of the well-known multi-objective evolutionary algorithm VEGA [6], which we call end-VEGA (elitist-non-dominated-VEGA). The extension, in its initial form introduced in [5], supplies the algorithm with the considerations related to elitism and non-dominance, lack of which is known to be its main drawback [7]. We also investigate the idea of utilizing ICA to initialize the population in the MOEA. The details of the modifications as well as a short theoretical background are given below.

## Methods

### Independent Component Analysis

Independent Component Analysis (ICA) is a signal processing technique originally developed to deal with the cocktail-party problem [8]. ICA is perhaps the most widely used method in Blind Source Separation (BSS) in various implementations and practical applications [9]. The basic idea in ICA is to represent a set of random variables using basis functions, which are as much statistically independent as possible. The Central Limit Theorem states that the distribution of a sum of independent random variables, under certain conditions, tends toward a Gaussian distribution. Thus a sum of two independent random variables usually has a distribution that is closer to Gaussian than any of the two original random variables. Therefore, the key concept in ICA is based on maximization of **non-Gaussianity **of the sources. There are various quantitative measures of non-Gaussianity, one of the most popular among which is **kurtosis **(or the fourth-order cumulant). One of the most popular ICA algorithms based on finding the local maximum of the absolute value of kurtosis is FastICA [10].

The open-source MATLAB package FastICA [11] was used as the implementation of the ICA algorithm in this project.

### Multi-Objective Evolutionary Algorithms

Many decision making or design problems involve optimization of multiple, rather than single, objectives simultaneously. In the case of a single objective, the goal is to obtain the best global minimum or maximum (depending on the nature of the given optimization problem), while with multi-objective optimization, there usually does not exist a single solution that is optimal with respect to all objectives. Therefore, the goal of multi-objective optimization is to find a *set *of solutions such that no other solution in the search space is superior to them when *all *objectives are considered. This set is known as **Pareto-optimal **or **non-dominated **set [7].

Since evolutionary algorithms (EA) work with a population of individuals, a number of Pareto-optimal solutions can be found in a single run. Therefore, an application of EAs to multi-objective optimization seems natural. The first practical MOEA implementation was the Vector Evaluated Genetic Algorithm (VEGA) proposed in [6]. Although it opened a new avenue in multi-objective optimization research, the algorithm seemed to have some serious limitations, at least partially due to the lack of considerations of **dominance **and **elitism **[7]. To deal with the first of the above considerations, a non-dominated sorting procedure was suggested in [12] and various implementations based on that idea of rewarding non-dominated solutions followed [13]. Elitism, in other words the notion that "elite" individuals cannot be expelled from the active gene-pool by worse individuals, has recently been indicated as a very important factor in MOEAs that can significantly improve their performance [14]. Both these aspects, while preserving the simplicity of implementation of the original VEGA, were taken into consideration in the design of the end-VEGA algorithm described here.

A C++ Evolutionary Algorithms library implemented by TGS was used for the MEOA experiments.

### Rough Sets

The theory of rough sets (RS) deals with the classificatory analysis of data tables [15]. The main idea behind it is the so-called **indiscernibility relation **that describes objects indistinguishable from one another. The indiscernibility relation induces a split of the universe *(i.e*., the set of all objects), by dividing it into disjoint equivalence classes, denoted as [x]_*B *_(for some object x described by a set of attributes *B*). These classes can be used to build new partitions of the universe. Partitions that are most often of interest are those that contain objects that belong to the same decision class. It may happen, however, that a concept cannot be defined in a crisp manner. The main goal of rough set analysis is to synthesize approximations of concepts from acquired data. Although it may be impossible to precisely define some concept *X*, we can approximate it using the information contained in *B *by constructing the *B*-**lower **and *B*-**upper approximations **of *X*, denoted by *BX *and *X *respectively, where, *BX *= {x|[x]_*B *_⊆ *X*} and *X *= {x|[x]_*B *_∩ *X *≠ ∅}. Only the objects in *BX *can be with certainty classified as members of *X*, based on the knowledge in *B*.

A rough set can be characterized numerically by the so-called **quality of classification**:



where *BX *is the lower approximation of *X*, *B*¬*X *is the lower approximation of the set of objects that do not belong to *X*, and *U *is the universe.

Another very important aspect of rough set analysis is data reduction by means of keeping only those attributes that preserve the indiscernibility relation and, consequently, the set approximation. The rejected attributes are redundant since their removal cannot worsen the classification. There are usually several such subsets of attributes and those that are minimal are called **reducts**. Finding a global minimal reduct (*i.e*., reduct with a minimal cardinality among all reducts) is an NP-hard problem. However, there are many heuristics (including utilization of genetic algorithms [16]) designed to deal with this problem.

RSL – The Rough Set Library [17] was used for the estimation of the RS-based fitness function measures in this project.

### ICA, RS, and MOEA-based Classificatory Decomposition

The main concept of classificatory decomposition (CD) was motivated by the hybridization of EAs with sparse coding with overcomplete bases (SCOB) introduced in [18]. Using this approach, the basis functions as well as the coefficients are being evolved by optimization of a fitness function that minimizes the reconstruction error and at the same time maximizes the sparseness of the basis function coding. This methodology produces a set of basis functions and a set of sparse (*i.e*., "as few as possible") coefficients. This may significantly reduce dimensionality of a given problem but, just as ICA, does not assure the classificatory usefulness of the resultant model.

In the approach proposed here, the sparseness term is replaced by a rough sets-derived data reduction-driven classification accuracy measure. This should assure that the result will be both "valid" (*i.e*., via the reconstruction constraint) and useful for the classification task. Furthermore, since the classification-related constituent also searches for a reduct, the classification is done with as few as possible basis functions. Finally, the single-objective EA utilized in the aforementioned technique is replaced by a multi-objective approach, in which the EA deals with the reconstruction error and classification accuracy, both at the same time [4].

Since the approach proposed here is based upon finding a solution satisfying two potentially conflicting goals *(i.e*., component-based reconstruction accuracy vs. classification accuracy), an application of MOEAs seems natural. In the experiments described here, we investigate a simple extension of VEGA, which supplies it with elitism and non-dominance, lack of which is known to be its main drawback. We call this extended algorithm end-VEGA (elitist-non-dominated-VEGA).

### end-VEGA

The main idea in VEGA is to randomly divide the population, in each generation, into equal subpopulations. Each subpopulation is assigned fitness based on a different objective function. Then, the crossover between the subpopulations is performed as with traditional EAs, with an introduction of random mutations.

As indicated earlier, VEGA has several quite significant limitations related to the lack of dominance and elitism. To address the former, we propose a simple approach based on multiplying the fitness of a given individual by the number of solutions that this individual is dominated by (plus 1 to ensure that the fitness function of a non-dominated solution is not multiplied by 0). Since the fitness function is being minimized in this project, the dominated solutions will be adequately penalized. To deal with the latter, we utilize the idea of an external sequential archive [14] to keep track of the best-so-far (*i.e*., non-dominated) solutions and to make sure that their genetic material is in the active gene-pool.

#### The general schema for the end-VEGA algorithm

The general schema for the application of end-VEGA for classificatory decomposition can be represented by the following pseudo-code:

t = 0;

P(t) := InitializePopulation();

A(t) := InitializeArchive();

while ( t < max. number of generations ) do

        [P_*rec*. _(t), P_*class*. _(t)] := DividePopulation ( P(t) );

        EvaluateFitness ( P_*rec*. _(t),  *f*_*RECONSTRUCTION *_);

        EvaluateFitness ( P_*class*. _(t),  *f*_*CLASSIFICATION *_);

        t := t + 1;

        P_*rec*. _(t) := Select( P_*rec*. _(t-1) );

        P_*class*. _(t) := Select( P_*class*. _(t-1) );

        P(t) := Crossover( P_*rec*. _(t), P_*class*. _(t), A(t) );

        Mutate ( P(t) );

        A(t) := GetNonDominated( P(t) ∪ A(t-1) );

end while;

#### Chromosome coding

**Figure 4:**
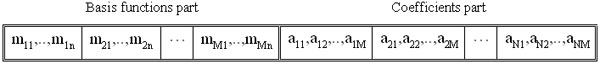
Chromosome coding

Each chromosome forms a complete solution to a given classificatory decomposition task and provides a description of both the set of basis functions and the coefficients for all the signals in the training data set. For example, for N signals with n samples each, and the task of finding M basis functions, the chromosome will be coded in the way presented in Fig. 4.

Each of the M basis functions has the length of the original input signal (*i.e*., n), and there are N vectors of coefficients (*i.e*., each vector corresponds to one signal in the training set) of dimensionality equal to the number of basis functions (*i.e*., each coefficient corresponds to one basis function).

#### Fitness evaluation: Reconstruction error

The measure employed in this project to calculate the distance between the original and the reconstructed signals is the well known 2-norm [19], referred to in signal processing as the signal energy-based measure, presented in (3).



where **x **represents the original signal, **M **is the matrix of basis functions, **a **is a set of coefficients, and *t *= 1..n, where n is the number of samples in the signal.

In order to deal with raw signals which can have large numbers as values (thus causing the energy-based distance measure to be large as well), a simple normalization of the energy-based measure by the energy of the original signal is proposed [4]:



Subsequently, the reconstruction error fitness function *f*_*REC *_for a chromosome *p *takes the following form:



where  is the normalized reconstruction error for the *i*^*th *^signal and N is the total number of the input signals.

#### Fitness evaluation: Classification accuracy and reduction in the number of coefficients and basis functions

The problem of maximizing the classificatory competence of the decomposition scheme, and at the same time reducing the number of computed basis functions, can be dealt with by the application of rough sets. In this project, the rough sets-based *quality of classification*, as presented in (2), is used for the purpose of estimating the classificatory aptitude.

The quality of classification is computed directly on the candidate reduct, which can be computed by any of the existing algorithms/heuristics. In this project, we are utilizing a variation of a simple greedy algorithm to compute a single candidate reduct only, as described by Johnson in [20].

Note that the main objective that deals with the classificatory capability of decomposition can actually be considered a bi-objective optimization problem itself. On one hand, we are looking for the best possible classification accuracy, but on the other, we want to use as few basis functions as possible. However, based on previous applications of EAs in the search for reducts, as described in [16], we decided to deal with it by minimizing a single-objective fitness function that is simply a summation of the classification error and the relative length of the reduct, as shown in (6).



where *p *is a given representative (*i.e*., chromosome), *L*(*R*) is the length of the potential reduct *R *(*i.e*., the number of attributes used in the representative), normalized by the total number of conditional (*i.e*., non-decisional/classificational) attributes M, and γ_*R *_is the *quality of classification *coefficient for the candidate reduct *R*.

An interesting question here is what to do with the coefficients (and the corresponding basis functions) that are not a part of the reduct. Since we are looking for the best possible classification accuracy, while using as few basis functions as possible, some mechanism capable of emphasizing the "important" coefficients/basis functions would be advisable. A solution to this problem is possible due to the application of the "hard" fitness computation idea, which allows the fitness function itself to introduce changes directly to the genetic material of the evaluated chromosome [2]. In this paper we propose to utilize a coefficients/basis functions annihilation approach, which simply zeroes-out the "not important" genetic material. The idea here is that if we remove the basis functions that are not vital in the classification process, the EA will improve the remaining basis functions in order to compensate for an increase in the reconstruction error.

## Results and Discussion

### Experimental data

**Figure 5:**
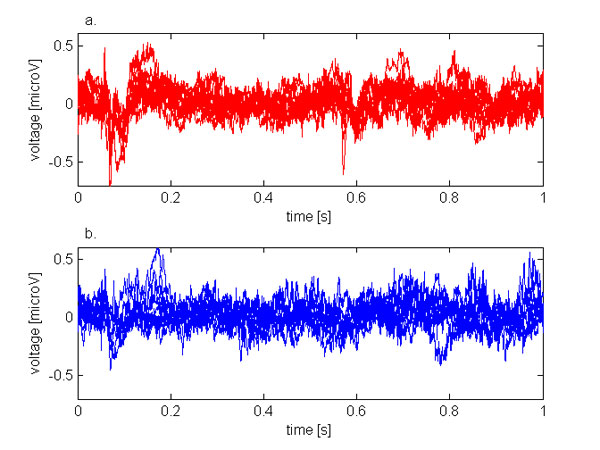
Input EPs for the unexposed (a) and exposed (b) animal

The dataset used in this study was derived from neurophysiological experiments performed at Arkansas State University [1]. In the experiments, recordings in the form of evoked potentials (EPs) of a duration of 1 second triggered by an auditory stimulus were collected from the cerebral cortex of two rats. One of the animals had been exposed to the cigarette smoke in utero (*i.e*., mother of the animal was exposed to cigarette smoke during pregnancy), while the other had not. The research problem here is to investigate how treatments (like nicotine) could alter responses to discrete stimuli. 10 signals were registered for the unexposed animal and 9 for the exposed one. The EPs were sampled at the rate of 7 kHz. The original signals for the unexposed and exposed rats are shown in Fig. 5.

### Analysis

**Figure 6:**
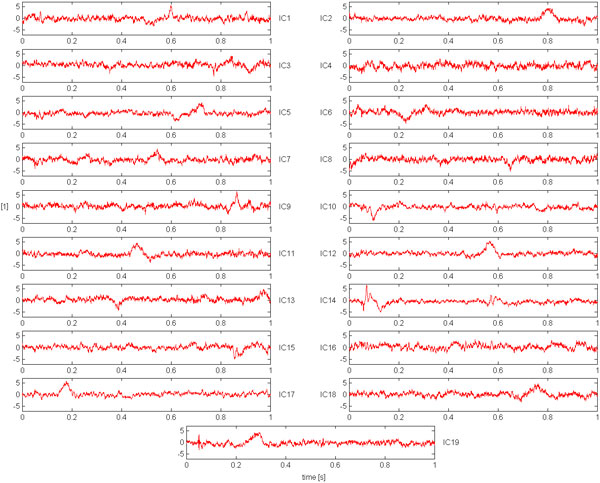
19 independent components computed using ICA

In the first step of the analysis described in this paper, the FastICA algorithm was utilized to compute the ICs to be used in the initial population in the MOEA. The algorithm yielded 19 ICs (shown in Fig. 6) along with the corresponding coefficients. As typical with ICA, the reconstruction was nearly perfect (see Table 1), but the entire set of 19 components had to be used to achieve that level of precision. Furthermore, the differentiation between the two EP classes (unexposed vs. exposed), based on all the corresponding coefficients, was not completely accurate (see Table 2). Finally, as the cardinality of the resultant set of ICs was the same as the number of the input signals, there was no indication as to which of the discovered independent components were significant for the underlying classification task.

In order to investigate the feasibility of the proposed approach, a number of MOEAs was launched simultaneously. The best results were obtained with the number of maximum possible generations set to 200 and the size of the population set to 30. Mutation probability was initialized with a small random value and was being adapted along the evolution process (*i.e*., increased by a small factor if no progress in the fitness functions was observed and reset when there was an improvement). Crossover probability was randomly determined in each generation (between 0% and 100%). Single-point crossover was utilized. All genetic operators were applied across both decision classes, as the ICA-derived initial ICs were computed for the entire dataset, without any additional information regarding the classification.

Due to the very limited available number of signals, the entire dataset of 19 EPs was used for both training and testing in our experiments. In the future, to assure a higher robustness of the response obtained from the classifier being created along the decomposition process, the data should be split into training and testing parts.

Several variants of ICA used to initialize the population in end-VEGA were tried. Both initialization of the full as well as a part of the population were simulated. In the first case, the changes in the basis functions can only be introduced by mutation, while in the second, some randomness is present from the beginning. The maximum allowable number of basis functions was set to 5, 10, or 19. In the first two cases, a random subset of 5 or 10 ICs (out of all 19) was chosen for each chromosome, and in the third, a permutation of all 19 ICs was used.

**Figure 7:**
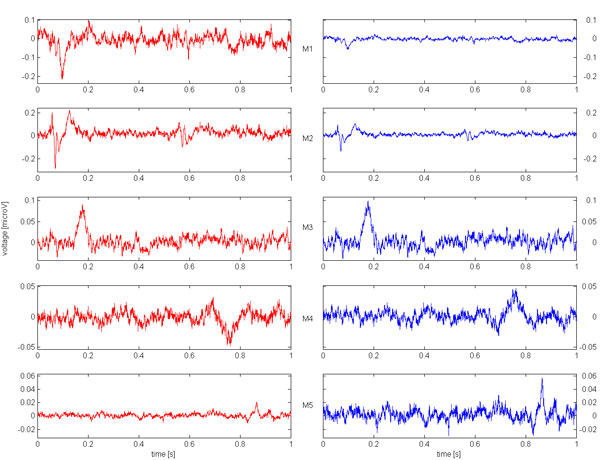
Selected averaged components for the unexposed (a) and exposed (b) animal

As an example, Fig. 7 presents a set of components, averaged for the unexposed and exposed animal separately, for 5 basis functions determined to be a 5-element reduct. The figure represents an average contribution of the basis functions in generation of the EPs in the unexposed and the exposed animal respectively.

Even a quick visual analysis of Fig. 7 reveals significant differences in how the sources are represented in the unexposed and the exposed rat. The dissimilarities can be simply expressed by amplitude variations (M1, M2, M3, M5), or can be as major as the sign reversal (M4). Further analysis of such phenomena can provide an interesting insight into the mechanisms behind the influence of nicotine on cortical neural activity. It is interesting to note that the general shape of the independent components computed by ICA has been preserved (*e.g*., compare M1 to IC10, M5 to IC9), but, as shown below, the reconstruction error is noticeably smaller thus indicating an improvement of the reduced set of resultant components.

The average reconstruction error was significantly improved as compared to the previous study [4], especially in the case of the full set of the ICs being used to initialize the MOEA. Note, however, that this set was still reduced to about 12, thus determining the ICs important for classification and at the same time "improving" them to account for the increase in the reconstruction error caused by removing the other 7 components, which were not classification-relevant according to the reduction algorithm. This impact of the improvement of the selected basis functions is clearly visible in Table 1, where we compare the reconstruction error of 5 components generated and "improved" by MOEA to a set of corresponding "non-improved" components taken directly from ICA. The collection of the corresponding original components taken directly from ICA was manually created by a visual comparison of the components shown in Fig. 7 to the ICA's basis functions presented in Fig. 6. As indicated earlier, the general shape of the independent components computed by ICA has been preserved, therefore it is easy to identify the prototypical ICs.

As for the classification accuracy, it was in most cases reasonably high (see Table 2) and the problems appeared to be related to 2 unexposed EPs being classified as exposed. The accuracy was statistically significantly improved as compared to ICA. The determined number of the basis functions required to preserve that accuracy (driven by the RS-based algorithm searching for a reduct) oscillated around 4, 6, and 12, for the maximum allowable number of 5, 10, and 19 of the basis functions respectively.

In order to assess and quantify the classification usefulness of the coefficients computed with the presented method and compare them with the ones obtained with ICA, a two-layer linear Artificial Neural Network (ANN) [21] was trained using the coefficients and tested for generalization. The ANN was trained with the leave-one-out training scheme: the training was performed N times, in each training instance coefficients that represent N-1 input signals were used for training, and the classifier was tested with the remaining 1 vector of the coefficients. A different vector of coefficients was used for testing in each training run. The testing error was measured as the difference between the ANN's output and the class label (desired output, set to -1 for the exposed group and 1 for the control group):

*E*_*i *_= *O*_*i *_- *Y*_*i*_,       (7)

where *O*_*i *_is the ANN's output for the *i*^*th *^vector of coefficients and *Y*_*i *_is the class label of the signal represented by the *i*^*th *^vector of the coefficients. The generalization is computed as the Mean Squared Error (MSE), using all testing errors:



Since ANN training strongly depends on the initial weight values (which are random), the leave-one-out training was repeated 50 times, and generalization errors were averaged. The resulting mean generalization errors are listed in Table 2, where the columns present the utilized decomposition method and its parameters, obtained generalization error, and significance of the fact that the decomposition method produces component coefficients that yield lower *E*_*MSE *_than those obtained with ICA, measured by the left-tailed t-test P-value.

The relatively large values of errors in Table 2 are due to the utilization of a linear classifier. The generalization error *E*_*MSE *_measures the average difference between the classifier's response and the desired response. Because a simple classifier was utilized, *E*_*MSE *_also measures how easy the classification of the vectors of coefficients is. Hence lower generalization error indicates that a given decomposition method produces components that are more useful for classification of the studied signals.

## Conclusion

This article presents a general framework for the methodology of classificatory decomposition of signals based on hybridization of independent component analysis, multi-objective evolutionary algorithms, and rough sets. In order to investigate the impact of the stimulus on the sources of neural activity, we designed a classification system that is capable of "predicting" if a given signal was registered under one or the other condition, solely based on the decomposition coefficients. Thus the relation between the stimuli and the sources can be analyzed. The preliminary results described here are very promising and further investigation of other MOEAs and/or RS-based classification accuracy measures should be pursued.

The incorporation of ICA-derived basis functions and coefficients as the starting point in the MOEA significantly improved the reconstruction error and more closely related the concept of classificatory decomposition to the traditional signal decomposition techniques. On the other hand, one of the main advantages of our approach is the fact that rather than solely relying on often unrealistic assumptions about statistical independence of sources, it generates a reduced set of components that are relevant in the light of a given classification problem itself.

The modifications in end-VEGA, although they improved the reconstruction slightly and sped up the overall convergence of the algorithm as compared to previous experiments, worked much better in tandem with ICA. In future research, it would be interesting to apply the results obtained using other decomposition approaches, *e.g*., Principal Component Analysis (PCA) [22] or Sparse Coding with Overcomplete Bases (SCOB) [23,24], as the initial population in MOEA.

## Authors' contributions

RB performed the underlying neurophysiological experiments. TGS and GMB implemented the testing software and performed the simulations. TGS, RB, GMB, MM, and AAP analyzed the results. All authors read and approved the final manuscript.

## Figures and Tables

**Table 1:** Comparison of the reconstruction errors. The error is calculated using the signal energy-based measure introduced in (5) for all 19 ICA's components (ICA), 5 classificatory decomposition components determined by the algorithm as important for classification and at the same time "improved" for reconstruction (CD 5), and the prototypical 5 components (determined by similarities in shape) taken directly from ICA without an "improvement" (ICA 5).

Signal no.	ICA	CD 5	ICA 5
1	3.28E-14	0.6835	1.2386
2	0.00E+00	0.6839	1.3235
3	1.17E-03	0.9051	1.3046
4	4.93E-03	0.4079	2.1672
5	1.14E-03	0.7817	1.1331
6	2.61E-03	0.3956	0.8999
7	2.55E-03	0.4523	1.0975
8	1.42E-03	0.4390	0.8135
9	3.64E-03	0.2983	1.3739
10	2.50E-04	0.4254	1.2926
11	3.74E-03	0.5594	2.0227
12	1.07E-03	0.6931	1.7776
13	1.04E-02	0.7806	1.8558
14	6.75E-03	0.7468	4.3669
15	1.29E-02	0.9559	1.0434
16	1.54E-03	0.7442	1.1452
17	9.60E-04	0.8155	1.2484
18	4.28E-04	0.4873	1.4637
19	1.78E-04	0.3161	0.6808

Average	5.37E-14	0.6090	1.4868

**Table 2:** Comparison of the generalization errors. The mean generalization errors (*E*_*MSE*_) are computed for the vectors of coefficients found by the presented classificatory decomposition method (CD) and ICA. The *P-value *represents the significance level of the fact that the presented method produces components that are more convenient for classification (*P-value *≤ 0.05 implies statistical significance). The errors were averaged over 10 trials for each CD method.

Method	*E*_*MSE*_	*P-value*
CD with max. no. of basis functions = 5	1.2817	< 0.0001
CD with max. no. of basis functions = 10	0.5605	< 0.0001
CD with max. no. of basis functions = 19	2.3545	1.0000
ICA	1.4186	
